# Mesostructural study on graphenic-based carbon prepared from coconut shells by heat treatment and liquid exfoliation

**DOI:** 10.1016/j.heliyon.2022.e09032

**Published:** 2022-02-26

**Authors:** Deril Ristiani, Retno Asih, Fahmi Astuti, Malik Anjelh Baqiya, Chonthicha Kaewhan, Sarayut Tunmee, Hideki Nakajima, Siriwat Soontaranon, Darminto

**Affiliations:** a Department of Physics, Faculty of Science and Data Analytics, Institut Teknologi Sepuluh Nopember, Surabaya 60111, Indonesia; b Synchrotron Light Research Institute, 111 University Venue, Muang District, Nakhon Ratcashima 30000, Thailand

**Keywords:** Carbon, Coconut shell, Graphenic, Mesostructure, Heat treatments, SAXS

## Abstract

In this study, the effect of heating temperature on the structure of graphenic-based carbon (GC) has been successfully investigated. A series of GC materials was prepared from coconut shells by a green synthesis method. The process includes heating at four temperatures (T = 400, 600, 800 and 1000 °C) followed by an exfoliation process assisted by hydrochloric acid (HCl). These materials were characterized by wide- and small-angle x-ray scattering (WAXS and SAXS), Fourier-transform infrared spectroscopy (FTIR), x-ray photoemission spectroscopy (XPS) and transmission electron microscopy (TEM). The WAXS analysis shows Braggs peaks corresponding to the reduced graphene oxide (rGO)-like phase. Investigations by FTIR and XPS methods show the presence of carbon-oxygen functional groups such as C=C (carbon with *sp*^2^ hybridization), C–C (carbon with *sp*^3^ hybridization), and C=O bonds. The *sp*^2^ bonds form a 2-dimensional (2D) network in hexagonal lattice, while carbon with *sp*^3^ bonds tends to form a 3-dimensional (3D) tetrahedral structure. The BET analysis revealed meso- and micro-pore structures in GC. Heating process reduces the specific surface area and increases pore size of GC. Moreover, increasing the heating temperature induces a decrease in radius of gyration (*R*_g_) and an increase in the formation of 2D structures in GC. The fitting results of SAXS profiles, proved by TEM and XPS, yielded the structure of GC containing the mixture of 2D and 3D structures. Thus, it is suggested that the GC has a mesostructure.

## 1 Introduction

Since the discovery of graphene by Novoselov and Geim, it has attracted huge interest in the science of carbon materials [1]. As a new material, the use of graphene is remarkable because of its superior mechanical, thermal, electric, and magnetic properties [2]. Graphene is a two-dimensional layered material consisting of carbon atoms arranged in a honeycomb lattice [3]. When oxidized, it forms graphene oxide (GO), in which various oxygen-containing functionalities are present [4]. Further, GO can be reduced to become reduced graphene oxide (rGO): a promising material for various applications, such as high capacity energy storage [5, 6], sensors [7], super capacitors [8], solar cell applications [9], hybrid electrocatalysts [10], high-performance electrode materials [11], microwave absorber [12, 13], and medical applications [14]. Due to potential applications of GC, it is important to understand its structure. Its physical and mechanical properties are suggested to strongly depend on its structure, including size and shape. For that reason, a more detailed investigation of the GC structure is necessary to obtain its expected properties.

Currently, an environmentally friendly starting material to produce graphene-based materials is more preferred. In nature, there are many sources of carbon, most of which originate from mining activities such as graphite, coal, and diamond as well as natural gas and liquid hydrocarbon sources (oil and gas). Carbon compounds from mining materials are categorized as non-renewable and non-environmentally friendly resources. Meanwhile, natural carbon sources are also available in the form of biomaterials derived from plants, either as products (bio-products) or waste (bio-waste), which are more environmentally friendly. An rGO-like structure has been found standing on natural carbon sources, coconut shell charcoal, by the facile synthesis, including the heating and the exfoliation processes [15, 16, 17, 18]. Differences in synthesis route and treatment provide a unique and different nature of the obtained rGO [2]. Various synthesis methods have been performed to yield rGO, such as epitaxial growth [19], chemical vapor deposition (CVD) [20], hydrothermal [21], and mechanical, thermal and chemical exfoliations [22]. Chemical exfoliation and thermal methods are the most widely used since they are easy to synthesize and controllable [23]. A. V. Dolbin et al. investigated the effect of the thermal reduction temperature on rGO and revealed that the reduction temperature affects the final oxygen content and creates structural damage by removing water and oxygen functionalities from the surface [24]. They also clearly state that high temperature treatments contribute to restore *sp*^2^ hybridized bonds. In addition, S. Farah et al. stated that the oxygen functionalities could be partially removed and create further defects by both thermal and chemical reduction methods [22]. Therefore, controlling the structure of graphene during its synthesis treatments is the key in preparing materials for many applications.

In the present work, graphenic-based carbon (GC) was produced using coconut shells as starting materials. Both the thermal and hydrochloric acid-assisted chemical exfoliation methods was applied for this production. It has been reported that the heating process, followed by the exfoliation process, is effective in exfoliating and breaking the van der Waals bonds between carbon layers [17, 24]. The use of hydrochloric acid has also been proven to reduce the particle size of graphene [17]. In this study, the heating process was carried out at temperatures ranging from 400 to 1000 °C to observe changes in the structure of the obtained GC. In a study conducted by M. A. Baqiya et al., the x-ray diffraction (XRD) and FTIR characterization show the formation of an rGO-like structure, and the presence of molecular bonds such as C=C, C–C, C–H, C–O, C=O, and O–H prove the formation of the rGO-like structure [15, 16, 17, 18]. Beyond XRD, here, WAXS measurement is performed to investigate the structure and phases of the obtained GC particles. WAXS is an x-ray technique typically employed to analyze the crystallinity in polymer systems [25, 26]. Graphene has been surface-characterized using some material characterization techniques such as transmission electron microscopy (TEM) and atomic force microscopy (AFM) [17, 27]. All these types of structural characterization only explain qualitatively and lack explanation regarding structural quantification. SAXS is a well-known quantitative technique that can complement AFM, TEM, and other surface characterization techniques [28, 29]. The SAXS technique can give an average value of the size of sheets. It has been used earlier for analyzing the structure of graphene-based particles prepared from coconut shell charcoal and revealed that the structure contains some particles formed as spherical and agglomerated plate-like flakes with a nano-sized radius [17]. However, structural quantification for bigger structures is not detailed yet. Therefore, a better model is proposed for detailing and analyzing the structure of GC.

## 2 Experimental

### 2.1 Materials

In this study, the materials used were old coconut shells and hydrochloric acid (HCl). Waste of old coconut shells was collected from local food market in Indonesia. The HCl (ACS reagent, 37%) is supplied from Merck India Ltd.

### 2.2 Synthesis of GC

**Figure 1:**
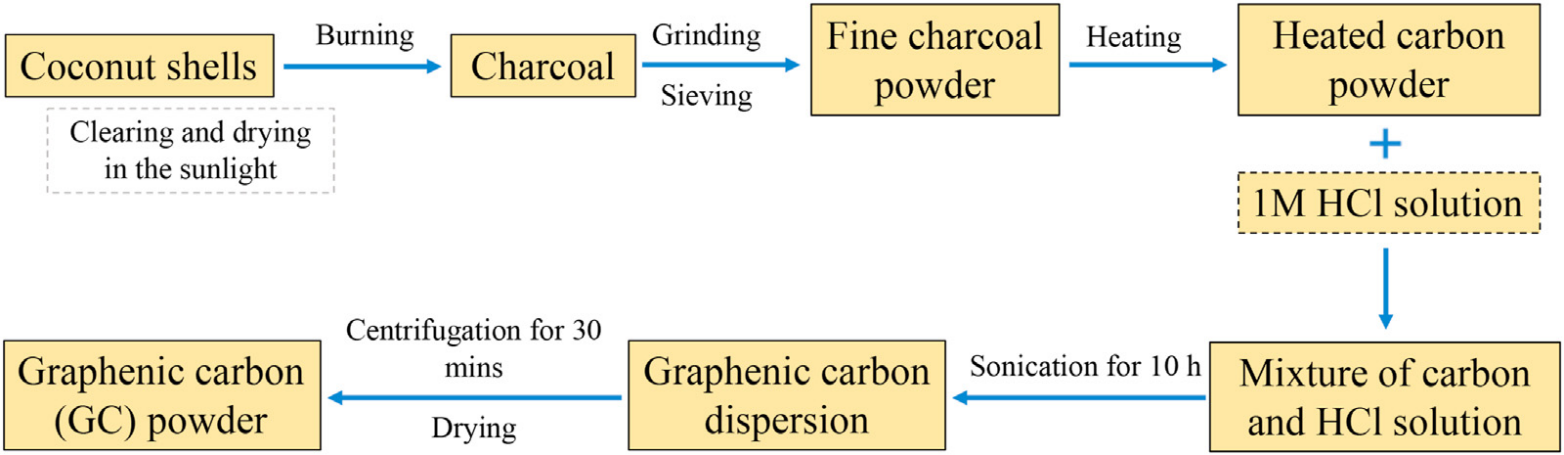
A schematic diagram of fabricating GC from old coconut shells using heat treatments and HCl-assisted exfoliation techniques.

Firstly, old coconut shells were cleaned, dried, and burned to obtain charcoal. The charcoal was then crushed and sieved to obtain fine charcoal powder with a homogeneous particle size. The charcoal powder was heated with temperature variations of 400, 600, 800, and 1000 °C for 5 h to obtain GC powder. A liquid-based exfoliation technique was used to release stacked GC sheets [15, 16, 17, 18]. This exfoliation process was assisted by HCl during bath sonication. The first step was to make a GC solution by dispersing 4 g of GC powder into 100 ml of 1M HCl solution. The solution then was stirred for 24 h at temperature of 80 °C using a magnetic stirrer. The solution obtained was directly ultra-sonicated for 10 h at room temperature. To separate the deposits from the liquid part, the solution was centrifuged for 30 min at 3000 rpm. The deposits were then dried using a hot plate at a temperature of 100 °C for 5 h. These powders are labeled as GC400, GC600, GC800, and GC1000 (GC means graphene-based carbon, and the numbers indicate heating temperatures). To aid comprehension, a simple schematic that summarizes the entire synthesis process is presented in Figure 1.

### 2.3 Characterization

Thermal analysis utilizing METTLER TOLEDO TGA (thermogravimetric analysis) was used to investigate the decomposition characteristics of coconut shells. Structural characterization of the GC was analyzed by WAXS diffraction patterns in the 2*θ* range of 10–55°. The WAXS patterns of the GC powders were collected at the Siam Photon Laboratory (SPL) of the Synchrotron Light Research Institute (SLRI), Thailand, using Beamline 1.3W. The WAXS patterns were displayed in two-dimensional (2D) scattering intensity maps, then converted and presented as scattering intensity *I*(*q*) vs. the angle between the incident and the diffracted x-ray (2*θ*). Fourier transform infrared spectroscopy (FTIR) measurements were conducted by the Shimadzu 8400S series in the wavenumber range of 400–4000 cm^−1^. The chemical composition and chemical bonding of the GC was characterized using the X-ray photoelectron spectroscopy (XPS) technique at Beamline 3.2Ua (BL3.2Ua) of the SLRI, Thailand. Raman spectroscopy (RAMAN iHR320 HORIBA) was also used to qualitatively assess the defects in the materials. This measurement is critical for determining the disorder structures present in carbon-based materials. The Brunauer-Emmett-Teller (BET) model was used to analyze the specific surface area and pore size distribution utilizing the Quantachrome Novatouch Lx4 instrument. The surface microstructure and morphology of GC was characterized using Scanning and Transmission Electron Microscopy (SEM, Zeiss EVO MA10 and TEM, Hitachi HT7700 operating at 120kV), respectively. In order to investigate the size and shape of the GC, Small angle x-ray scattering (SAXS) measurements were carried out in the beamline BL1.3 W of the SLRI. SAXS, an important non-destructive tool for characterizing materials, has electron density fluctuation on the length scale of about 1–100 nm [17].

## 3 Results and discussion

**Figure 2:**
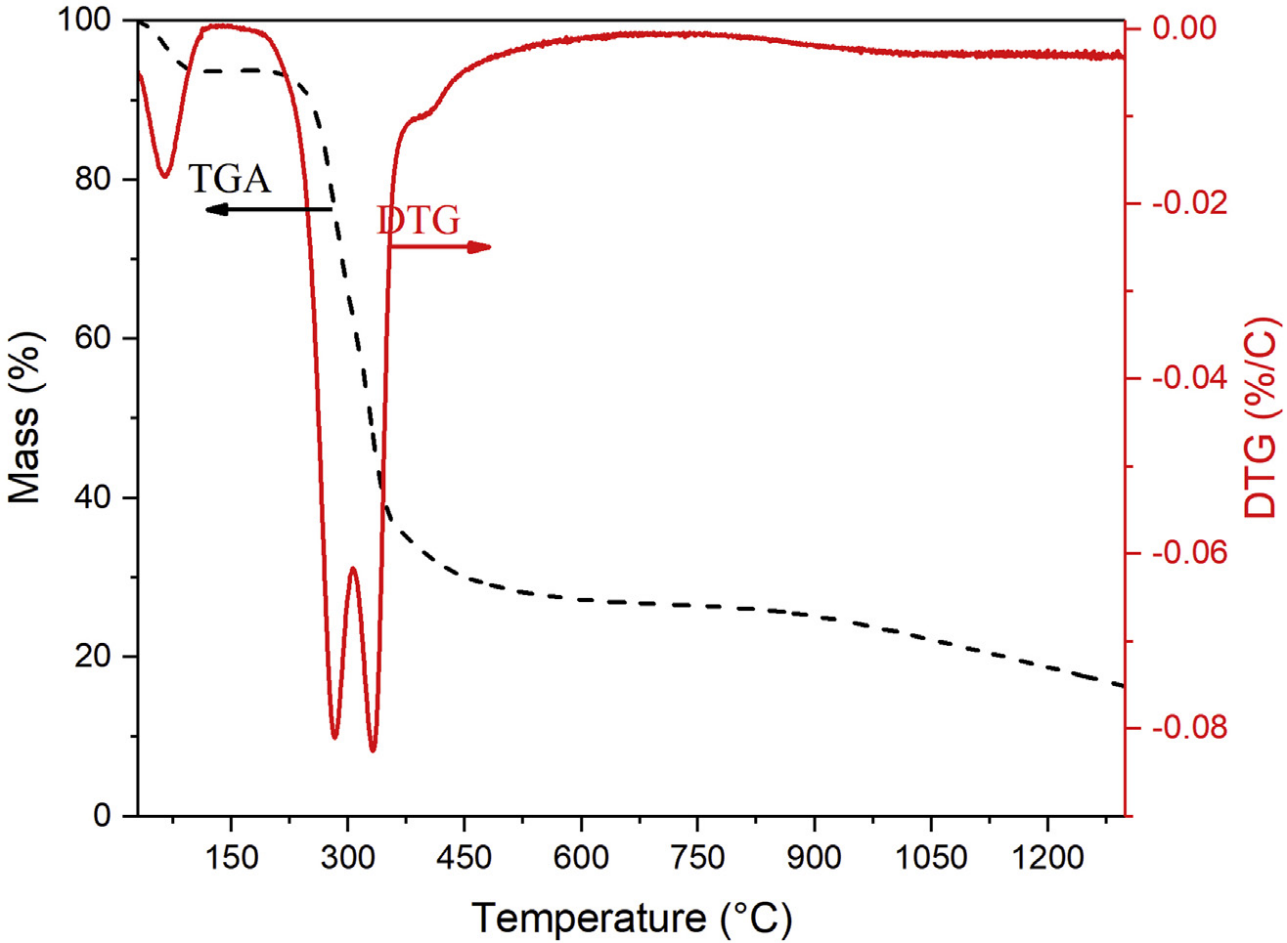
TGA and its corresponding DTG curve of coconut shells.

The thermal analysis of coconut shells using TGA is shown in Figure 2. It can be used to estimate the thermal decomposition behavior of biomass as a function of weight reduction (TGA curve) and its first derivative (DTG - derivative thermogravimetric curve). A minor peak was observed in the DTG curve at temperatures below 100 °C, which corresponds to the release of intrinsic water [30, 31], resulting in an approximate mass loss of 5.7%. A significant amount of mass loss (∼45.26 %) occurs between 200 and 400 °C. Generally, heat degradation of biomass occurs as a result of hemicellulose, cellulose, and lignin decomposition [32, 33]. Two distinct peaks at 283.50 and 336.78 °C can be associated with the decomposition of hemicellulose and cellulose content in coconut shells, respectively [30] which results in a weight loss of approximately 22.87% and 22.39%. Slow degradation occurs at temperatures greater than 400 °C and continues up to 1300 °C with a weight loss of 16.36%. This can be attributed to lignin decomposition and the degradation of certain oxygen functional groups [30].

**Figure 3:**
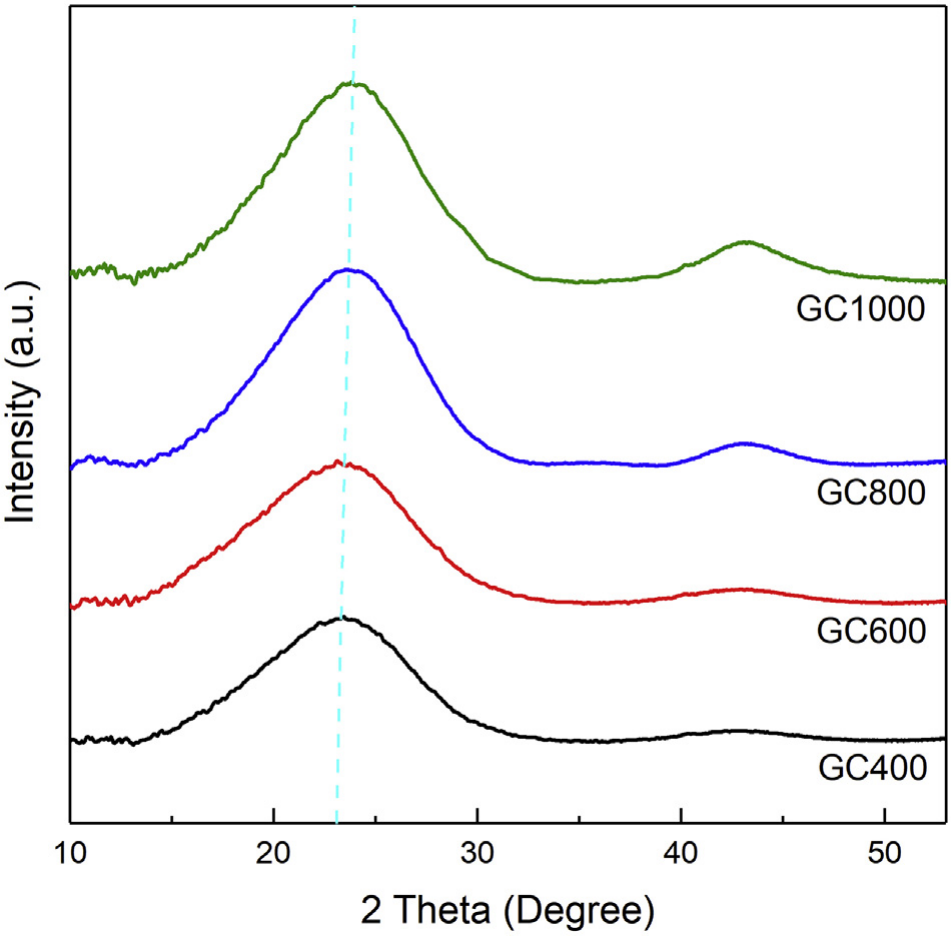
WAXS spectra of the GC samples.

Figure 3 shows WAXS patterns of GC. WAXS is a diffraction-based technique that can be used to investigate crystal structure and orientation and to verify the average distance between graphenic layers [34, 35].

The WAXS pattern shows that the appearance of two broad peaks at positions ∼23° and ∼43° correspond to the (002) plane and the (100) plane, respectively. It indicates the characteristics of a pristine rGO phase. The broad peaks may be attributed to the arrangement of the graphene layers, which tend to be random along the stacking direction of graphene sheets. This implies that the GC has peeled off into one or only few graphene layers with a lattice spacing of 0.39 nm. This is significantly greater than the lattice spacing in graphite with a value of approximately 0.33 nm. It indicates that the graphene layers are well-separated and not stacked as in graphite [36]. Furthermore, as temperature increases, a more intense peak at (100) plane is observed. This could be associated with a reduction of oxygen functional groups and defects in the sample, which would be further confirmed using FTIR analysis.

**Figure 4:**
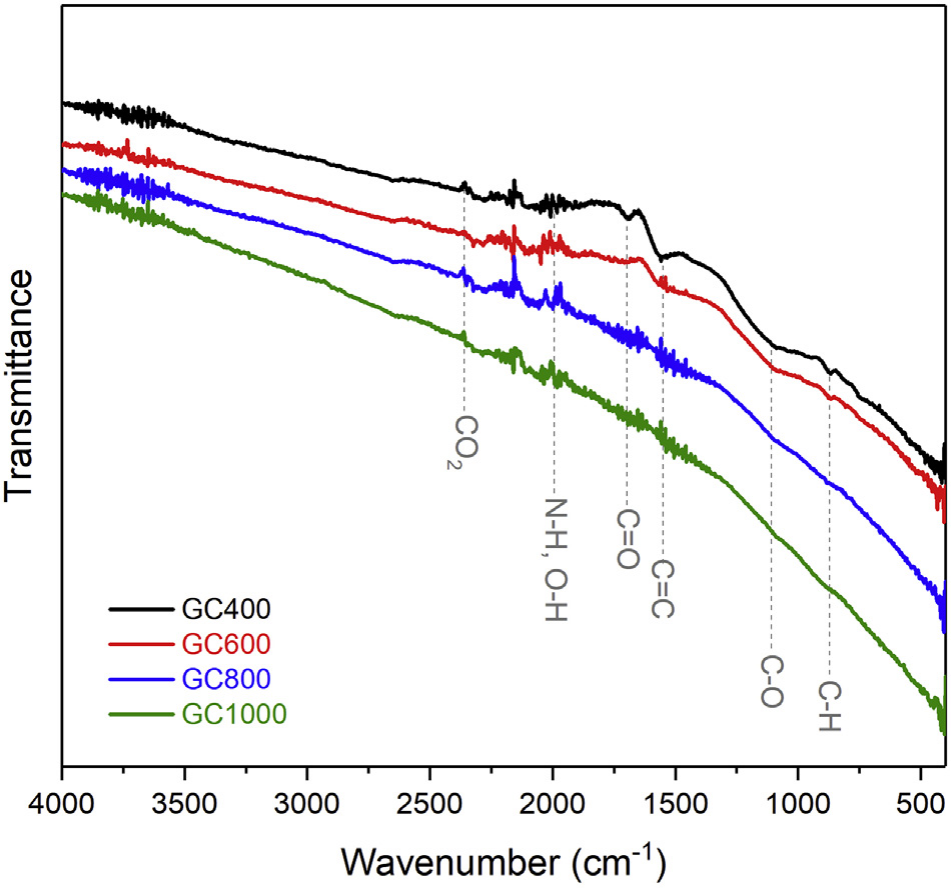
FTIR spectra of the GC samples.

FTIR spectra of GC are presented in Figure 4, showing the presence of similar types of molecular bonds/functional groups in all of the samples. The molecular bonds that appear are indicated by the presence of the transmittance peaks of the FTIR pattern. Each molecular bond has a different wave number based on the ability of the molecular bonds to vibrate and absorb energy from the infrared spectrum. The results of the FTIR pattern of all samples qualitatively show that the main molecular bonds on rGO were identified, such as C=C and C–O bonds. Both of them bond together to form the hexagonal structure of the carbon atoms which are arranged into the rGO layer. Transmittance peaks in the FTIR spectrum of GC observed at ∼1696 cm^−1^ that corresponds to C=O/carbonyl stretching vibrations, those at ∼1550 cm^−1^ that denotes C=C stretching vibrations, ∼1107 cm^−1^ that represents stretching vibrations from C–O, and ∼871 cm^−1^ that is assigned as C–H/hydrocarbyl vibrations [17, 37]. It is noticeable that the absorption intensity of some oxygen functional groups decreases with increasing the heating temperature. This confirms the WAXS results, in which as the temperature increases, the detected peak of the (100) plane gets more intense. Thus, it can be assumed that the more intense peak in (100) plane is due to the decreased oxygen functional group. A deeper analysis using x-ray photoemission spectroscopy (XPS) will be quite useful to confirm the moieties present in the samples.

**Figure 5:**
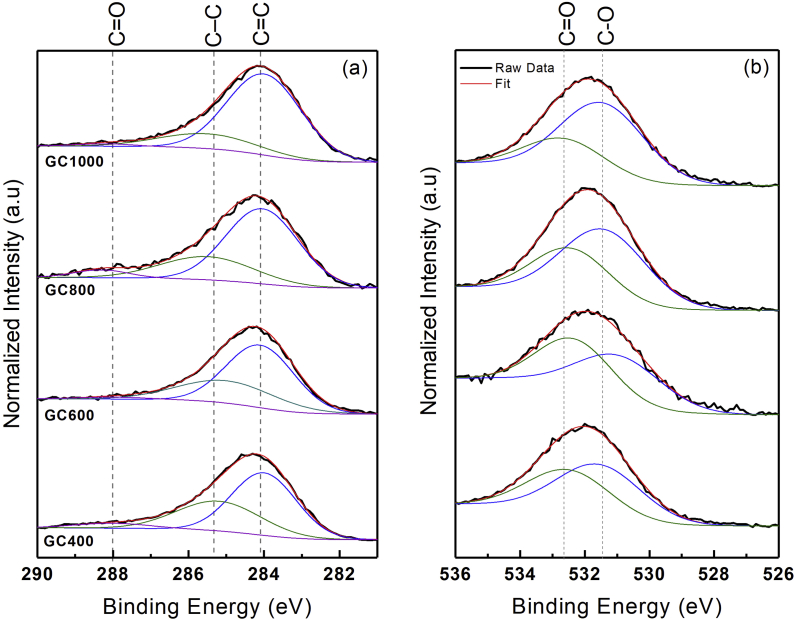
High-resolution XPS spectra of the (a) C1s (b) O1s core level peak.

XPS is a spectroscopic technique used for quantitative surface analysis. It can provide chemical information on the sample, such as elemental composition, impurities, empirical formula and electronic state of the element [38, 39]. In this study, XPS was performed to measure the chemical bonding and *sp*^2^/*sp*^3^ ratio in GC. The high-resolution C1s and O1s spectra of the obtained GC are presented in Figure 5 (a) and (b), respectively. The C1s and O1s spectra were fitted by the Gaussian function and the Shirley background correction. Deconvolution was performed using Microsoft Excel equipped with macros software. The C1s spectra were deconvoluted into three component peaks attributed to carbon with *sp*^2^ hybridization (C=C) at ∼284 eV, carbon with *sp*^3^ hybridization (C–C) at ∼285 eV, and the carbonyl carbon (C=O) at ∼288 eV [40, 41]. The O1s spectral peaks show two intense peaks at ∼532.7 eV ascribed to oxygen double-bonded to carbon (C=O) and oxygen single-bonded to carbon appearing at ∼531.5 eV.

**Figure 6:**
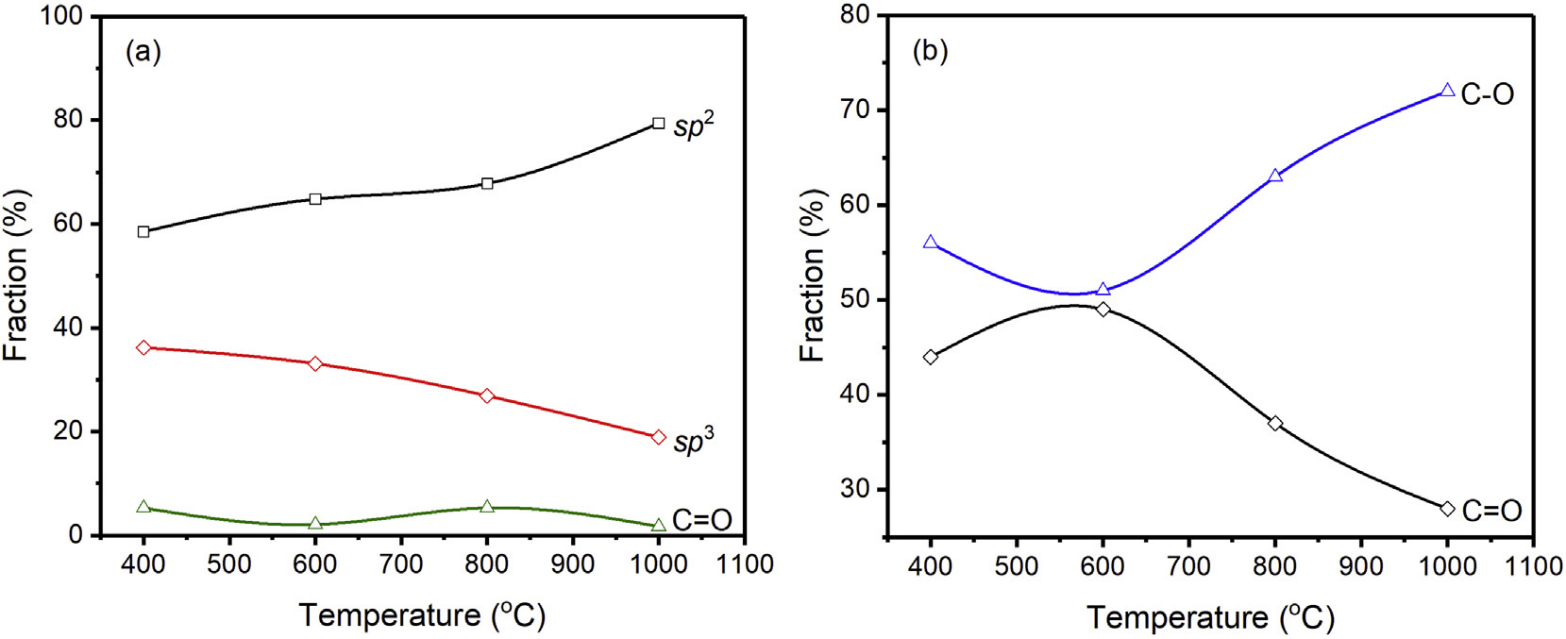
Carbon constituents of the deconvoluted (a) C1s and (b) O1s core level peak. The use of solid lines aims for eye guidance.

Figure 6 displays the results of the deconvolution of the C1s and O1s spectra. The bonds present in the GC samples consist of C=C, C–C, and C=O bonds. Thus, the GC prepared from coconut shell charcoal is dominated by carbon bonds. The content of C=C regions/*sp*^2^ hybridization increases by increasing the reduction temperature. This is possible when the heating temperature increases—then the energy generated for the formation of C=C will also increase, so that the level of *sp*^2^ hybridization increases by increasing temperatures. The C–C component decreases with the reduction temperature, pointing to a lower quantity of *sp*^3^ hybridization. The percentage of oxygen-containing bonds (C=O) tends to decrease. It suggests that the heating process can cause the oxygen bonds to break and form reduced graphene oxide. The releasing of oxygen bonds is known to induce defects in the GC structure, which will be described further in the explanation of Raman spectroscopy. When compared to the XPS spectra from Ref [42, 43], it is obvious that graphene derivatives are mostly composed of C=C *sp*^2^ bonds, with modest contributions from C–C *sp*^3^, C–O, and C=O bonds. The report also mentions the same phenomenon as this study, namely a decrease in the contribution of the C=O group after reduction, including chemical and thermal reduction [42, 43]. Furthermore, the *sp*^2^/*sp*^3^ ratio increases significantly, indicating that the majority of the oxygen-containing functional groups are removed due to the heat treatment. A pristine rGO is *sp*^2^ bonded, which forms a 2-dimensional series in a hexagonal lattice, while carbon with *sp*^3^ bonds tends to form a 3-dimensional tetrahedral structure. In this work, the GC was produced with a mixed *sp*^2^ and *sp*^3^ bonds, which also means a mixture of 2D and 3D structures. Thus, it is suggested that the GC has a mesostructure, which is probably much different from the nanostructure.

**Figure 7:**
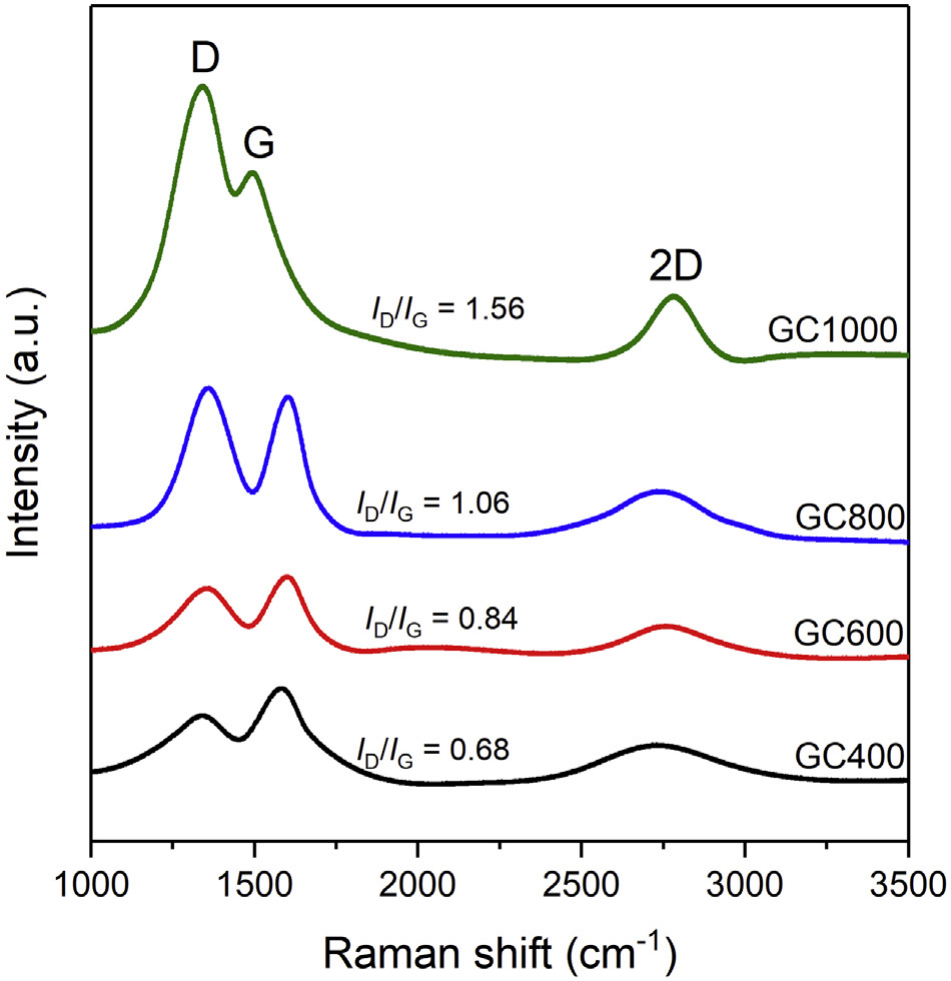
Raman spectra of the GC samples.

GC samples were also subjected to Raman spectroscopy in order to further understand their graphitic and defect structures. The Raman spectra of the GC samples are displayed in Figure 7. It shows two main peaks that are characteristic of the D- and G-bands. The D- and G-peaks were observed at 1344 and 1503 cm^−1^, respectively. Specifically, the D band is linked with defects in the structure (*A*_1g_ mode), while the G band is associated with *E*_2g_ phonon scattering due to the *sp*^2^ hybrid structure [44, 45]. Additionally, the intensity ratio of the D and G peaks (*I*_D_/*I*_G_) in all samples was calculated, which indicates that the *I*_D_/*I*_G_ increases as the heating temperature increases. Therefore, GC1000 has the highest *I*_D_/*I*_G_ value, indicating that it has more defects or disorders than the other samples [46]. These defects can be in the form of vacancy defects due to the release of oxygen bonds in the structure, as well as line defects and carbon adatoms [47, 48]. The presence of an apparent 2D-peak at 2750 cm^−1^ indicates that a two-dimensional structure has formed on the GC samples [17, 46] which is getting more intense as the temperature increases. In comparison to standard graphene, which is synthesized from graphite using a modified Hummers' method, graphene oxide has an obvious D-peak with an intensity proportional to the G-peak [42, 44, 46]. It differs from pristine graphene, which shows a lower intensity of the D peak. It implies that graphene oxide has more structural defects than pristine graphene [46]. A similar issue occurs with GC samples, in which the GC exhibits increasing structural disorders due to defects, oxygen functional groups, and reconstruction of 2D structures as the heating temperature increases. The GC samples exhibit a higher defect rate when compared to derived standard graphene materials reported by A. Ganguly et al. [46].

The BET surface area analysis technique is extremely effective at determining the porosity and specific surface area of materials. The Barrett-Joyner-Halenda (BJH) method was used to estimate the pore size distribution [49, 50]. The result reveals that the number of pores in GC400 is significantly higher than that in GC1000, with the majority of the pores measuring greater than 2 nm, although there are also some pores that are 1–2 nm in size. On the other hand, GC1000 contains pores of a size greater than 10 nm, but in relatively lower quantities than GC400. As a result, the total pore volume of GC400 and GC1000 were calculated to be 0.09 and 0.04 cc/g, respectively. The pore distribution explicitly indicates that the GC is composed of meso- and micro-pore structures [51, 52, 53, 54]. The GC400 shows greater specific surface area and total pore volume than that of the GC1000. From these results, it can be concluded that the heating process results in an increase in pore size and a decrease in the specific surface area and total pore volume of the material, as shown in Table 1. This tendency is consistent with XRD data, which shows that crystallization increases as the heating temperature rises. According to the XPS data described earlier, increasing the heating temperature results in an increase in *sp*^2^ bonds and a decrease in *sp*^3^ bonds. The *sp*^3^ bonds can be associated with the diamond-*like* structure, while *sp*^2^ bonds can be associated with the graphite-*like* structure. According to a study conducted by P.T. Moseley et al. [55], diamond has a higher density than graphite, which means it has the opposite porosity feature. This is consistent with the results of this study, which indicate that the higher *sp*^2^ content in the sample results in greater pore size. The GC1000 contains higher *sp*^2^ bonds than the GC400, resulting in a greater pore size.

**Table 1:** Total pore volume, pore diameter and specific surface area of the GC400 and GC1000 samples examined by BET technique.

Sample	Total Pore Volume (cc/g)	Pore Diameter (nm)	Specific Surface Area (m^2^/g)
GC400	0.09	1.69	122.8
GC1000	0.04	13.20	77.4

**Figure 8:**
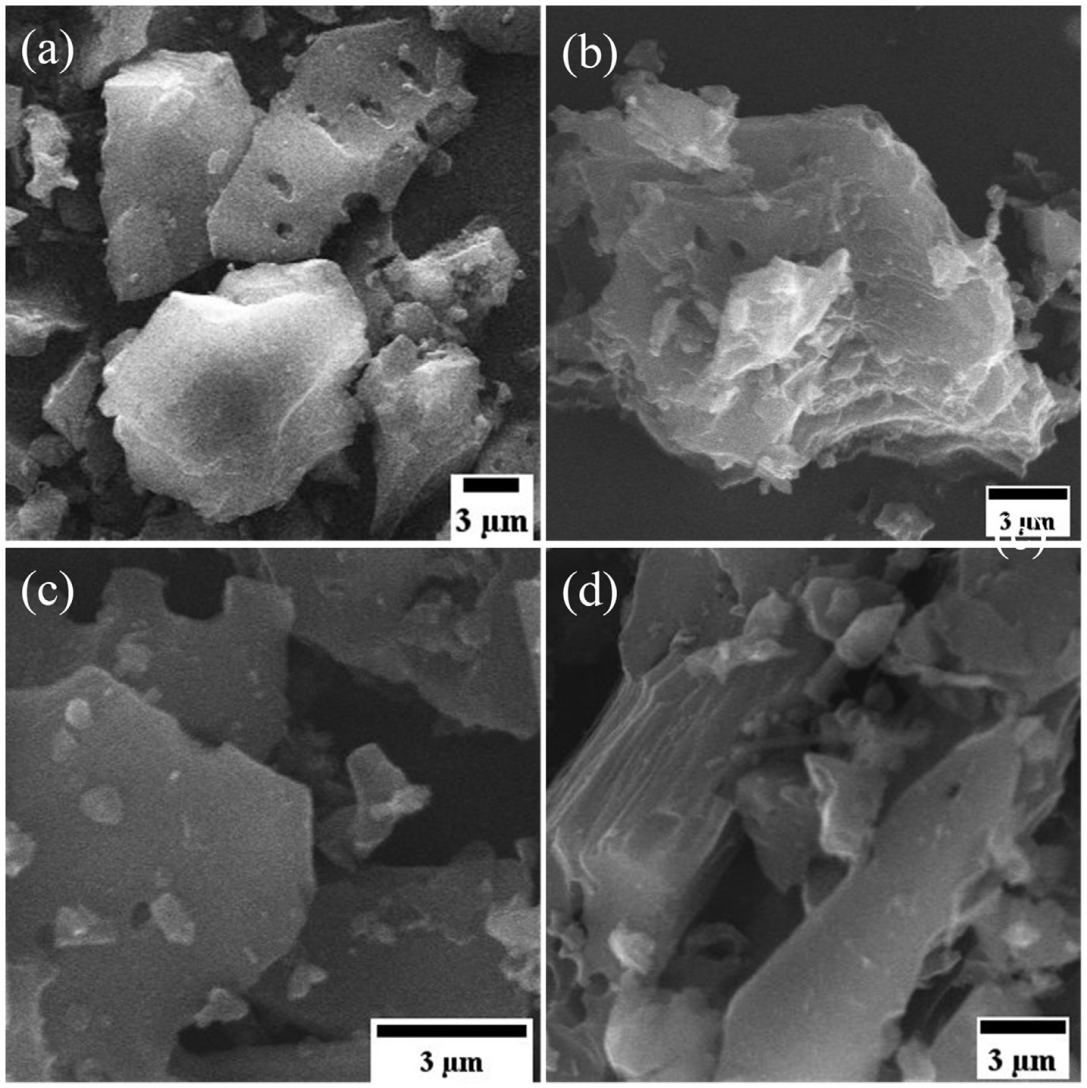
SEM image of the exfoliated GC heated at: (a) 400 °C (b) 600 °C (c) 800 °C, and (d) 1000 °C.

**Figure 9:**
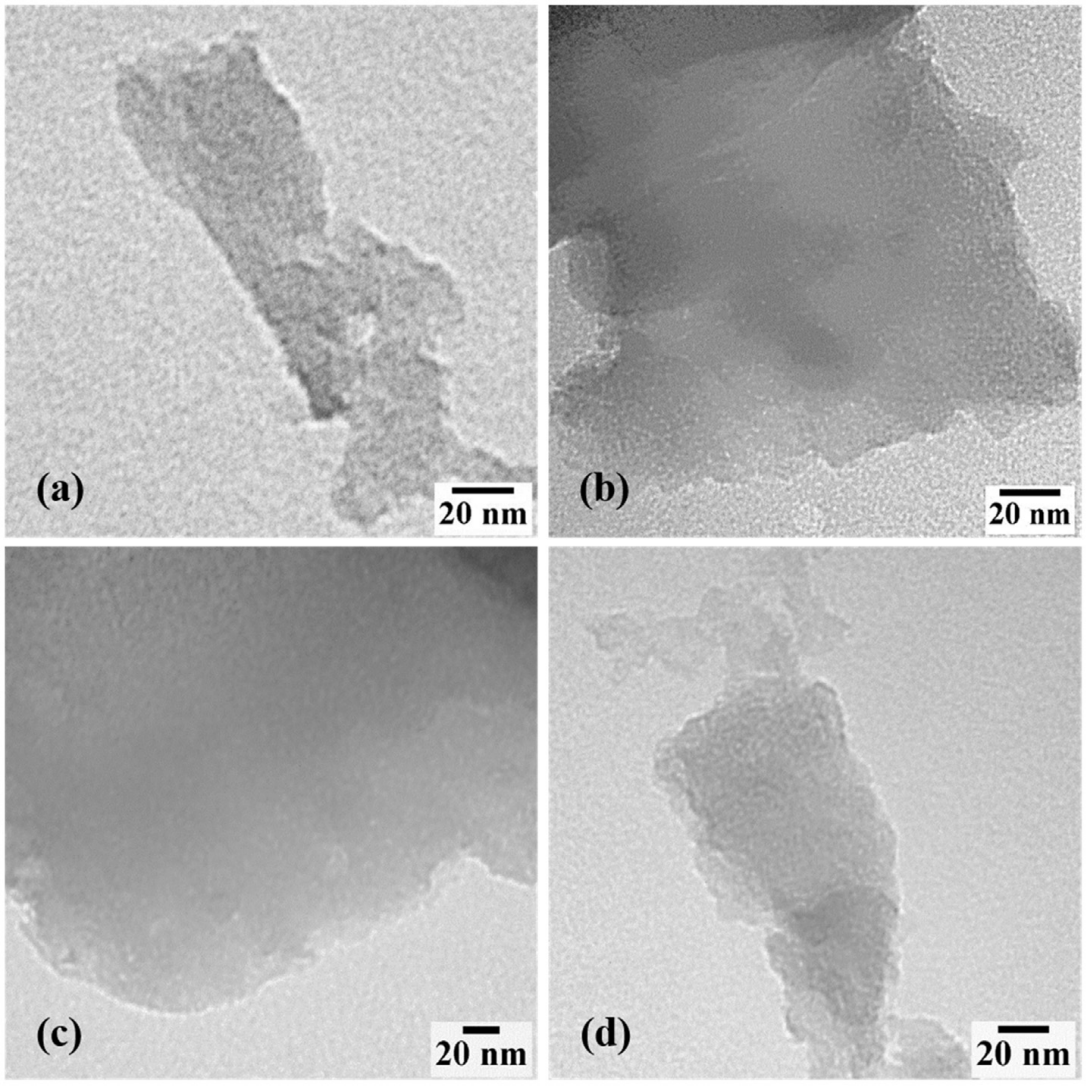
TEM image of the exfoliated GC heated at: (a) 400 °C (b) 600 °C (c) 800 °C, and (d) 1000 °C.

The surface morphologies of the GC samples are probed using SEM and low-resolution TEM images. The SEM photographs of GC samples (Figure 8) demonstrate the typical structure of GC, which is formed of crumpled spherical shapes along with sheet-like shapes. The GC samples that were heated to a higher temperature were less wrinkled. This could be because the heating increased the spaces between the graphenic layers, resulting in an unfolding structure. Thus, it can be inferred that heat treatments have the potential to effectively exfoliate the obtained GC. TEM images also reveal that the GC consists of graphenic flakes with sizes ranging up to a few nanometers, as presented in Figure 9(a –d). It also shows a surface corrugation in some parts that relate to a localized intrinsic strain due to the loading of oxygen functional groups at the graphenic sheets [56]. A comparable microstructure has also been discovered in standard graphene materials by F. J. Sonia and et al. [42], which exhibit the characteristics of folded and crumpled morphology. The corresponding TEM images in their study further reveal that the standard graphene materials are formed of wrinkled graphene sheets. This is consistent with our observations in this work. Furthermore, at a closer view of the TEM images, the basal planes of all GC samples did not show any significant morphological differences. The GC1000 has more crumples and damages at the edges probably because the water removal during the thermal expansion process is harder at 1000 °C and destroys the carbon structure more intensively [24]. However, this fact did not result in a significant change in the XRD pattern. It is clear that the high temperature contributes to the *sp*^2^ network restoration.

**Figure 10:**
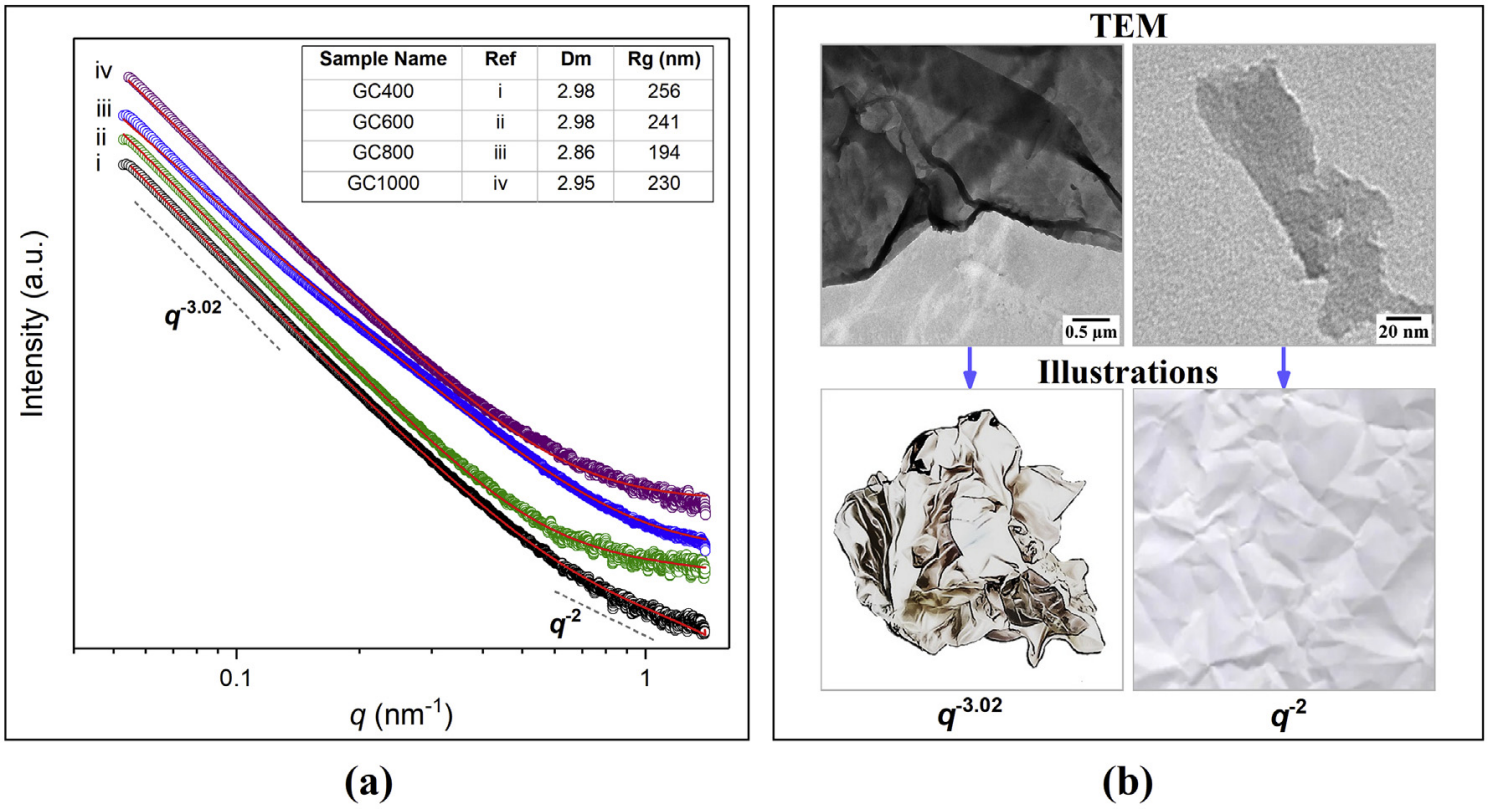
(a) SAXS profiles of the GC samples, and (b) illustrations showing the fractal dimensions compared to TEM images.

Beyond TEM observation, SAXS was employed to quantitatively determine the particle size and shape (form factor) of GC samples. Figure 10 shows the intensities of X-rays scattered by a sample as a function of the scattering angle [34, 57]. SAXS uses the basic principle of Bragg's law in small-angle scattering; the source of high-energy x-rays hit a particle in the sample, then the scattering is detected at a detector with a certain distance. Here, the x-ray energy used in SAXS characterization was 9 keV with a sample-to-detector distance (SDD) of 4.3 m. The use of SDD is very important during the measurement process because it affects the scattering angle used to determine the range of the scattering vector (*q*). The scattering vector is related to the measured particle size. The long SDD will produce a low *q* range (<1nm^−1^), while the low *q* range represents a larger particle. Meanwhile, the short SDD will produce a high *q* range (>1nm^−1^) and represent a smaller particle [58, 59]. In contrast to the SAXS measurements carried out in many previous studies [17, 60, 61], in this study, the measurements were carried out on powder-based samples instead of colloidal states. M. A. Baqiya et al. conducted a preliminary structural study using SAXS with a colloidal solution of graphene as a substance [17]. Compared to our results, the scattering patterns of powder-based samples show steady data compared to that of colloidal-based samples, of which the scattering data are more fluctuating, especially in the lower *q* range. Moreover, they stated that the Beaucage model is successful in modeling the scattering pattern of the exfoliated graphene-based particle. They also suggested that the exfoliated graphene-based particle formed a spherical shape and agglomerated plate-like flakes with a radius in the nanometer order [17]. As an in-depth analysis of SAXS that includes large particles, this work proposes to focus on the analysis using the mass fractal model [62, 63]. The mass fractal model is considered more suitable in representing the structure of the graphene-based materials prepared from coconut shell charcoal. T. M. McCoy and S. Pradhan stated that the mass fractal function is suitable for quantifying the 2- and 3-dimensional structures present in graphene-based structure [60, 61].

In Figure 10, the scattering profile for GC samples shows two structural levels. At each level, the GC samples show a mass fractal regime which illustrates both 3-dimensional structure in lower *q* range and 2-dimensional structure in higher *q* range. The illustration model in Figure 10 is used to describe the two structural levels stated. The first level illustrates a crumpled paper sheet in 3-dimensional form. When a crumpled 3-dimensional lump is stretched, the series of 2-dimensional sheets will be seen. These 2-dimensional sheets are called persistence sheets, as they are the primary structure arranging the building block of the crumpled 3D sheet [61].

In order to model the scattering GC samples, a mass fractal model was preferred. The mass fractal model estimates the scattering of inhomogeneous substances according to the power law as *I* (*q*) ∝ *q*^−α^, which allows for the determination of the fractal dimensions (*D*_m_) of the material. *D*_m_ can provide information about the complexity of the structure, where higher *D*_m_ indicates higher complexity [60, 62, 64]. The information about the fractal dimensionality can be obtained by determining the slope of the SAXS scattering intensity profile in the low *q* range as power law function [63]. 3D objects show the slope *α* = 3 or higher, while 2D objects show the slope *α* = 2. The observed slope for GC400, GC600, GC800, and GC1000 is in the range of 3.02–3.14, indicating the presence of aggregates or 3D objects [29]. N. Singh et al. in their report combined SANS and SAXS profiles to analyze the structure of graphene materials [65]. The SANS and SAXS profiles shown are similar to the SAXS profiles for the GC samples. The scattering experiment shows the presence of branching of the rGO cluster with the incorporation of nanoparticles. The fitting results show that the graphene fractal dimensions range from 2.1 to 3.0 depending on the preparation process [65]. Figure 10 presents the successful fitting curve of the GC samples using mass fractal model, as written in Eq. (1). This model was used to examine the structural quantification of GC samples.

(1) $$I\;\left(q\right)=\;\frac{\sin\left[\left(D_{m}-1\right)\;tan^{-1}\left(q\xi \right)\right]}{\left(D_{m}-1\right)q\xi {\left(1+q^{2}\xi^{2}\right)}^{\left(D_{m}-1\right)/2}}$$

where $\xi^{2}=2R_{g}^{2}/D_{m}\left(D_{m}+1\right)\;$, *I*(*q*) describes the scattering intensity as a function of the wave vector *q*, *D*_m_ defines the dimension of mass fractal, which must be a value between 0 and 6, and describes the fractal complexity. The parameter *ξ* is the cut-off length, which is a measure of the linear size of the aggregate or cluster proportional to the radius of gyration *R*_g_. *R*_g_ is the radius of gyration of the aggregate or cluster [60].

TEM images show a good agreement with the SAXS observation. It shows that the GC is composed of crumpled-like particles randomly arranged on the surfaces and tending to form the fractal structure with a size in the order of 200 nm. The size of the gyration radius (*R*_g_) of the particles and the shape of the particles were determined quantitatively through curve fitting using the Sasfit program. This process aims to quantitatively determine the size of the particle radius and the shape of its dimensions by matching the curve of the selected model with the curve of the measurement data. Table inserts in Figure 10 display the *R*_g_ and *D*_m_ of each material calculated using the mass fractal equation. It shows that the particles have a rough shape and are irregularly arranged. Here, the results reveal the change in radius of gyration with respect to heating temperatures. *R*_g_ tends to decrease with the increase in heating temperatures in the GC powders, meaning that there is a combination of 2D and 3D structures.

## 4 Conclusions

In summary, the GC synthesized from old coconut shells, using thermal and chemical exfoliation techniques, has been successfully prepared. The WAXS analysis shows Braggs peaks corresponding to rGO single phase, with the main broad peak appearing at 2*θ* ∼ 23^o^ corresponding to the (002) plane and less intensity peak at 2*θ* ∼ 43° corresponding to the (100) plane. The FTIR and XPS investigations reveal that the main bonds in the GC samples are C=C (carbon with *sp*^2^ hybridization), C–C (carbon with *sp*^3^ hybridization), and C=O bonds. As the temperature increases, the oxygen-containing bonds decrease and are suggested to create defects in the GC structure along with the reconstruction of the 2D structure. Raman spectroscopy further demonstrates that as the heating temperature increases, the GC exhibits increased structural disorder due to defects, oxygen functional groups, and reformation of 2D structures. Qualitatively, TEM images shows unique sheet-like smooth waves and crumpled surface with the size up to 250 nm. The result is in good agreement with the result of SAXS analysis, in which the *R*_g_ obtained is suggested to have a radius in the order of 200 nm. The SAXS fitting reveals that the structure of GC contains a mixture of 2D and 3D structures, which is confirmed by TEM and XPS. As a final point, the GC powder suggests a mesostructure. Due to their superior physical properties and comprehensive structural characterizations, the obtained GC is expected to contribute in potential applications, such as in the fields of microwave absorption devices and lithium ion battery cathodes.

## Declarations

### Author contribution statement

Deril Ristiani: Conceived and designed the experiments; Performed the experiments, Wrote the paper.

Retno Asih, Malik Anjelh Baqiya: Analyzed and interpreted the data; Wrote the paper.

Fahmi Astuti: Contributed reagents, materials, analysis tools or data.

Chonthicha Kaewhan, Sarayut Tunmee, Hideki Nakajima, Siriwat Soontaranon: Performed the experiments; Analyzed and interpreted the data.

Darminto: Conceived and designed the experiments; Wrote the paper.

### Funding statement

This work was supported by Kementerian Riset Teknologi Dan Pendidikan Tinggi Republik Indonesia (1286/PKS/ITS/2020) and the scholarship granted by the PMDSU Program (D.R.).

### Data availability statement

Data included in article/supplementary material/referenced in article.

### Declaration of interests statement

The authors declare no conflict of interest.

### Additional information

No additional information is available for this paper.
